# The state of artificial intelligence-based FDA-approved medical devices and algorithms: an online database

**DOI:** 10.1038/s41746-020-00324-0

**Published:** 2020-09-11

**Authors:** Stan Benjamens, Pranavsingh Dhunnoo, Bertalan Meskó

**Affiliations:** 1 Department of Surgery, University Medical Center Groningen, University of Groningen, Groningen, The Netherlands; 2 Medical Imaging Center, University Medical Center Groningen, University of Groningen, Groningen, The Netherlands; 3 The Medical Futurist Institute, Budapest, Hungary; 4 Department of Behavioural Sciences, Semmelweis University, Budapest, Hungary

**Keywords:** Health services, Outcomes research

## Abstract

At the beginning of the artificial intelligence (AI)/machine learning (ML) era, the expectations are high, and experts foresee that AI/ML shows potential for diagnosing, managing and treating a wide variety of medical conditions. However, the obstacles for implementation of AI/ML in daily clinical practice are numerous, especially regarding the regulation of these technologies. Therefore, we provide an insight into the currently available AI/ML-based medical devices and algorithms that have been approved by the US Food & Drugs Administration (FDA). We aimed to raise awareness of the importance of regulatory bodies, clearly stating whether a medical device is AI/ML based or not. Cross-checking and validating all approvals, we identified 64 AI/ML based, FDA approved medical devices and algorithms. Out of those, only 29 (45%) mentioned any AI/ML-related expressions in the official FDA announcement. The majority (85.9%) was approved by the FDA with a 510(k) clearance, while 8 (12.5%) received de novo pathway clearance and one (1.6%) premarket approval (PMA) clearance. Most of these technologies, notably 30 (46.9%), 16 (25.0%), and 10 (15.6%) were developed for the fields of Radiology, Cardiology and Internal Medicine/General Practice respectively. We have launched the first comprehensive and open access database of strictly AI/ML-based medical technologies that have been approved by the FDA. The database will be constantly updated.

## Introduction

The 2010s has brought a rise in the number of studies and papers discussing the role of artificial intelligence (AI) and machine learning (ML) in medicine and healthcare (AI/ML). The number of life science papers describing AI/ML rose from 596 in 2010 to 12,422 in 2019. While we are at the beginning of the AI/ML era, the expectations are high and experts foresee that AI/ML shows potential for diagnosing, managing and treating a wide variety of medical conditions^1^.

Indeed, AI/ML-based technologies have been shown to support several medical specialties from radiology^2^ and oncology^3^ to ophthalmology^4^ and general medical decision-making^5^. ML models have been shown to reduce waiting times^6^; improve medication adherence^7^; customize insulin dosages^8^; or help interpret magnetic resonance images^9^, among others.

Despite its promise, the obstacles for implementation of AI/ML in daily clinical practice are numerous^10^. These include issues with transparency surrounding these software programs, the inherent bias in the data they are fed with and how secure they are. A crucial element shaping these obstacles is regulating such technologies. The very use of the term AI requires further clarification, as multiple subtypes have been proposed, and its meaning can be vague. For the sake of further investments and the public image, companies tend to overuse the term AI, when in fact they have developed algorithms which are not AI/ML-based per se.

We classify a technology as AI/ML based if official FDA announcements, communications by the company or other publicly available information resources used the expressions ‘deep learning,’ ‘machine learning,’ ‘deep neural networks,’ ‘artificial intelligence,’ and/or ‘AI’ to describe the technology^11^. For simplicity, we use the term “AI/ML-based” to denote these technologies in this paper.With the increasing expertise and attention on AI/ML in the medical field, the opportunities and possible implications of its use are the topics of an ongoing debate^12^. A crucial element in this implementation debate is regulating such technologies.

As studies about AI’s role in medicine show, its use cases and companies developing such technologies have been skyrocketing, regulatory bodies, such as the US Food & Drugs Administration (FDA) and the European Medicine Agency (EMA), have tried to tackle its regulation and implementation. As the FDA has shown leadership regarding the adoption of AI/ML-based medical technologies, with a specific framework for AI/ML-based algorithms, we chose it as an example for further analysis^13^.

Because of the high-risk nature of these medical devices and the unknown consequences of using AI/ML for medical decision-making and data analysis, the FDA has stringent regulatory requirements for medical device licensing. Developers of AI/ML-based medical devices and algorithms have to go through rigorous processes that are time and resource consuming. This can be considered pivotal as a barrier for the introduction of AI/ML in medicine.

Before medical hardware or software is legally made available in the US market, the parent company has to submit it to the FDA for evaluation. For medically oriented AI/ML-based algorithms, the regulatory body has three levels of clearance, namely, 510(k)^14^, premarket approval^15^ and the de novo pathway^16^, each of which needs specific criteria to be fulfilled in order to be granted (Table 1).

**Table 1:** Descriptions of the types of FDA approvals for AI/ML-based medical technologies.

Level of FDA clearance	Description
510(k) clearance	A 510(k) clearance for an algorithm is granted when it has been shown to be at least as safe and effective as another similar, legally marketed algorithm. The submitter seeking this clearance must provide substantial proof of equivalence in their application. Without an approval of being substantially equivalent to the other algorithm, the one pending approval cannot be legally marketed.
Premarket approval	Premarket approval is issued to algorithms for Class III medical devices. The latter are those that can have a large impact on human health and as such, their evaluation undergo more thorough scientific and regulatory processes to determine their safety and effectiveness. In order to approve an application, the FDA determines that the device’s safety and effectiveness is supported by satisfactory scientific evidence. Upon approval, the applicant can proceed with marketing the product.
de novo pathway	Regarding the de novo classification, it is used to classify those novel medical devices for which there are no legally marketed counterparts, but which offer adequate safety and effectiveness with general controls. The FDA performs a risk-based assessment of the device in question before approval and allowing the device to be marketed.

For the development and marketing of medical algorithms, the FDA’s stringent regulatory requirements pose important challenges to the companies developing them. Before, every new product had to go through the regulatory process. However, as companies update their algorithms on a much shorter time scale, namely in days, the FDA has realized that this process might become impossible to maintain. Therefore, the FDA started to consider “a total product lifecycle-based regulatory framework for these technologies that would allow for modifications to be made from real-world learning and adaptation, while still ensuring that the safety and effectiveness of the software as a medical device is maintained”^13^.

To date, the FDA has cleared or approved several medical devices using “locked” algorithms. They define a “locked” algorithm as an algorithm that provides the same result each time the same input is applied to it and does not change. However, many recent medical devices, especially when AI/ML based, use algorithms that change and can adapt over time; these are described by the FDA as adaptive algorithms, for which current regulatory frameworks were not designed^17^. The power of these AI/ML-based algorithms lies within the ability to continuously learn, where change to the algorithm might only be realized after the device or software has been distributed for use and could learn from real-world experience.

An attempt to solve this issue was proposed in the FDA’s proposed regulatory framework from 2019^18^, that “elaborates on a potential approach to premarket review for artificial intelligence and ML-driven software modifications”^13^. It is recognized by the FDA that these adaptive algorithms require a total product lifecycle (TPLC) regulatory approach, enabling rapid cycle of product improvement with effective safeguards.

This TPLC approach is based on the Digital Health Software Precertification (Pre-Cert) Program^19^, allowing for the evaluation of Software as a Medical Device (SaMD) products throughout the lifecycle.

However, the FDA does not ask companies to categorize their technology as AI/ML based when in fact it is. And some companies mention that their technology is AI/ML based in the announcement of the FDA approval or the specific ML method they used, but others do not. We witnessed this issue while working on this paper ourselves.

At this moment, the evaluation of the processes for approval and implementation is hampered by a lack of clarity on the approval of AI/ML-based medical devices and algorithms, as FDA announcements do not clearly state the use of these methods. Moreover, the search engine of the FDA’s website does not allow users to perform specific search queries in FDA announcements and summaries, thus hampering the accessibility of the database. It can be expected from regulatory bodies to provide a clear description of such devices; create and maintain a clear database, allowing proper search queries, to assess the implementation of new techniques. The FDA, like other regulatory agencies, has not done any of these yet.

The purpose of this paper therefore was threefold: (1) to provide an insight into the currently available AI/ML-based medical devices and algorithms that have been approved by the FDA; (2) to create an up-to-date database of FDA approvals in this field that welcomes submissions and might serve as the database that the FDA should have; and (3) to raise awareness of the importance of regulatory bodies clearly stating whether a medical device is AI/ML based.

In line with this aim, we performed a systematic web-based search for announcements of FDA approvals of AI/ML-based medical devices and algorithms and cross-checked all approvals on FDA.gov resulting in an open access and continuously expandable database.

To our knowledge, this is the first comprehensive database of strictly AI/ML-based medical technologies, in all medical specialties, that have been approved by the FDA. We also suggest a threshold and definition of what defines a medical technology as AI/ML based.

## Results

### Cross-checked and validated medical devices and algorithms

Cross-checking and validation of all announcements resulted in a database with 64 AI/ML based, FDA-approved medical devices and algorithms. We decided to include only those 29 devices in our further analysis that met the criteria of being considered an AI/ML-based technology in the related official FDA announcements (Table 2). For the other 35 devices, online sources other than the FDA marked them as AI/ML-based technologies. A comprehensive overview is provided in the online open access database.

**Table 2:** Database of the 29 FDA-approved, AI/ML-based medical technologies.

#	Name of device or algorithm	Name of parent company	Short description	FDA approval number	Type of FDA approval	Mention of algorithm in announcement	Date	Medical specialty	Secondary medical specialty
1	Arterys Cardio DL	Arterys Inc.	Software analyzing cardiovascular images from MR	K163253	510(k) premarket notification	Deep learning	2016 11	Radiology	Cardiology
2	EnsoSleep	EnsoData, Inc.	Diagnosis of sleep disorders	K162627	510(k) premarket notification	Automated algorithm	2017 03	Neurology	
3	Arterys Oncology DL	Arterys Inc.	Medical diagnostic application	K173542	510(k) premarket notification	Deep learning	2017 11	Radiology	Oncology
4	Idx	IDx LLC.	Detection of diabetic retinopathy	DEN180001	de novo pathway	AI	2018 01	Ophthalmology	
5	ContaCT	Viz.AI.	Stroke detection on CT	DEN170073	de novo pathway	AI	2018 02	Radiology	Neurology
6	OsteoDetect	Imagen Technologies, Inc.	X-ray wrist fracture diagnosis	DEN180005	de novo pathway	Deep learning	2018 02	Radiology	Emergency Medicine
7	Guardian Connect System	Medtronic	Predicting blood glucose changes	P160007	PMA	AI	2018 03	Endocrinology	
8	EchoMD Automated Ejection Fraction Software	Bay Labs, Inc.	Echocardiogram analysis	K173780	510(k) premarket notification	Machine learning	2018 05	Radiology	Cardiology
9	DreaMed	DreaMed Diabetes, Ltd.	Managing Type 1 diabetes	DEN170043	de novo pathway	AI	2018 06	Endocrinology	
10	BriefCase	Aidoc Medical, Ltd.	Triage and diagnosis of time sensitive patients	K180647	510(k) premarket notification	Deep learning	2018 07	Radiology	Emergency Medicine
11	ProFound™ AI Software V2.1	iCAD, Inc.	Breast density via mammography	K191994	510(k) premarket notification	Deep learning	2018 07	Radiology	Oncology
12	SubtlePET	Subtle Medical, Inc.	Radiology image processing software	K182336	510(k) premarket notification	Deep neural network-based algorithm	2018 8	Radiology	
13	Arterys MICA	Arterys Inc.	Liver and lung cancer diagnosis on CT and MRI	K182034	510(k) premarket notification	AI	2018 09	Radiology	Oncology
14	AI-ECG Platform	Shenzhen Carewell Electronics., Ltd.	ECG analysis support	K180432	510(k) premarket notification	AI-ECG	2018 09	Cardiology	
15	Accipiolx	MaxQ-Al Ltd.	Acute intracranial hemorrhage triage algorithm	K182177	510(k) premarket notification	Artificial intelligence algorithm	2018 10	Radiology	Neurology
16	icobrain	icometrix NV	MRI brain interpretation	K181939	510(k) premarket notification	Machine learning and deep learning	2018 10	Radiology	Neurology
17	FerriSmart Analysis System	Resonance Health Analysis Service Pty Ltd.	Measure liver iron concentration	K182218	510(k) premarket notification	Artificial intelligence	2018 11	Internal Medicine	
18	cmTriage	CureMetrix, Inc.	Mammogram workflow	K183285	510(k) premarket notification	Artificial intelligence algorithm	2019 03	Radiology	Oncology
19	Deep Learning Image Reconstruction	GE Medical Systems, LLC.	CT image reconstruction	K183202	510(k) premarket notification	Deep learning	2019 04	Radiology	
20	HealthPNX	Zebra Medical Vision Ltd.	Chest X-Ray assessment pneumothorax	K190362	510(k) premarket notification	Artificial intelligence	2019 05	Radiology	Emergency Medicine
21	Advanced Intelligent Clear-IQ Engine (AiCE)	Canon Medical Systems Corporation	Noise reduction algorithm	K183046	510(k) premarket notification	Deep Convolutional Neural Network	2019 06	Radiology	
22	SubtleMR	Subtle Medical, Inc.	Radiology image processing software	K191688	510(k) premarket notification	Convulutional neural network	2019 7	Radiology	
23	AI-Rad Companion (Pulmonary)	Siemens Medical Solutions USA, Inc.	CT image reconstruction - pulmonary	K183271	510(k) premarket notification	Deep learning	2019 07	Radiology	
24	Critical Care Suite	GE Medical Systems, LLC.	Chest X-Ray assessment pneumothorax	K183182	510(k) premarket notification	Artificial intelligence algorithms	2019 08	Radiology	Emergency Medicine
25	AI-Rad Companion (Cardiovascular)	Siemens Medical Solutions USA, Inc.	CT image reconstruction - cardiovascular	K183268	510(k) premarket notification	Deep learning	2019 09	Radiology	
26	EchoGo Core	Ultromics Ltd.	Quantification and reporting of results of cardiovascular function	K191171	510(k) premarket notification	Machine learning-based algorithms	2019 11	Cardiology	Radiology
27	TransparaTM	Screenpoint Medical B.V.	Mammogram workflow	K192287	510(k) premarket notification	Machine learning components	2019 12	Radiology	Oncology
28	QuantX	Quantitative Insights, Inc.	Radiological software for lesions suspicious for cancer	DEN170022	de novo pathway	Artificial intelligence algorithm	2020 01	Radiology	Oncology
29	Eko Analysis Software	Eko Devices Inc.	Cardiac Monitor	K192004	510(k) premarket notification	Artificial neural network	2020 01	Cardiology	

**Fig. 1:**
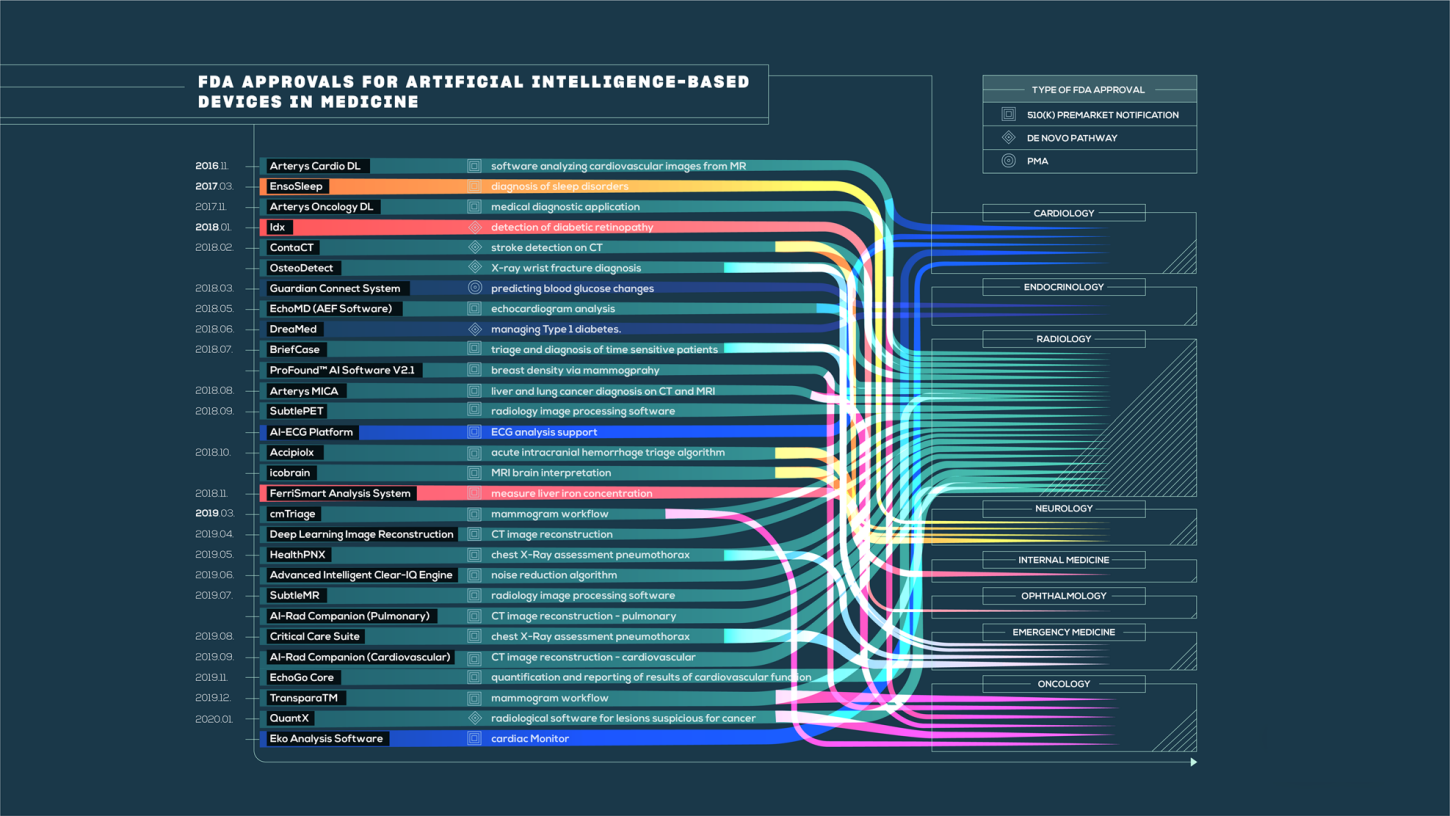
An infographic about the 29 FDA-approved, AI/ML-based medical technologies. The devices have features such as date and type of FDA approval; name of the device, its short description and which primary and secondary medical specialty it is related to.

A short overview of these medical devices and algorithms can be found in the infographic (Fig. 1) and a detailed overview, with subsequent directions to the official FDA announcements, is provided in the online open access database.

Of these medical devices and algorithms, the vast majority (*n* = 23, 79.3%) was approved by the FDA with a 510(k) clearance, while 5 (17.2%) received de novo pathway clearance and one (3.4%) received PMA clearance.

The first FDA approval was granted in the year 2016, with three approvals at the end of the year 2017. Most FDA approvals were granted in the year 2018, with 13 (44.8%) approvals, while 10 (34.4%) and 2 (6.9%) approvals were granted in 2019 and 2020 up until February, respectively.

The two main medical specialties with AI/ML-based medical innovations are Radiology and Cardiology, with 21 (72.4%) and 4 (13.8%) FDA approved medical devices and algorithms respectively. The remaining medical devices and algorithms can be grouped as focusing on internal medicine/endocrinology, neurology, ophthalmology, emergency medicine, and oncology.

The medical field of radiology is the trendsetter regarding FDA-approved medical devices and algorithms, with the introduction of AI/ML-based solutions for worldwide applied image reading software. Examples are the three algorithms for Arterys Inc., Arterys Cardio DL, Arterys Oncology DL and Arterys MICA, which are connected to the workflow Picture Archiving and Communication Systems from main vendors as Siemens Healthineers AG (Germany) and GE Healthcare (USA)^20^. Six out of these 21 algorithms can be applied in the field of oncology, with three focusing on mammography analyses (ProFound™ AI Software V2.1, cmTriage and TransparaTM) and three others on CT-based lesion detection (Arterys Oncology DL, Arterys MICA and QuantX). This is followed by two algorithms focusing on brain image analyses, with innovations for stroke and hemorrhage detection (ContaCT, Accipiolx, and icobrain), and six algorithms to improve image processing, with noise and radiation dosage reduction (SubtlePET, Deep Learning Image Reconstruction, Advanced Intelligent Clear-IQ Engine, SubtleMR, AI-Rad Companion (Pulmonary) and AI-Rad Companion (Cardiovascular)). Another four algorithms focusing on acute care, with two algorithms for the assessment of pneumothorax (HealthPNX and Critical Care Suite), one focusing on wrist fracture diagnosis (OsteoDetect) and the Aidoc Medical BriefCase system for triage of head, spine, and chest injuries. The final two algorithms in this specialty can be applied for cardiovascular assessments, focusing on the assessment of the heart ejection fraction (EchoMD AEF Software and EchoGo Core).

Cardiology is another category with major advancements, resulting in four FDA-approved medical devices and algorithms. Most investment goes to innovations for the detection of cardiac rhythm abnormalities, with FDA approval for the AI-ECG Platform and Eko Analysis Software. The other two algorithms overlap with the field of Radiology, being EchoMD AEF software and EchoGo Core.

With diabetes affecting a significant part of society, innovations to manage blood glucose levels were highly warranted. The first steps were made with the introduction of the Guardian Connect System by Medtronic and the DreaMed Diabetes system (DreaMed Diabetes Ltd)^21^. AI/ML-based interpretation of laboratory results was also introduced for the field of Internal Medicine, with the FerriSmart Analysis System (Resonance Health Analysis Service Pty Ltd) for liver iron concentration assessment.

To increase access to early eye disease detection, one company introduced an AI/ML-based algorithm for the interpretation of ophthalmology tests, being Idx (IDx LLC) for detection of diabetic retinopathy^22^.

There are also some devices and algorithms related to neurology, with broad overlap with the field of Radiology. In addition to these overlapping algorithms, EnsoSleep was introduced for the diagnosis of sleep disorders.

### Additional medical devices and algorithms

For these 35 medical devices, the application of AI/ML has not been confirmed by the official FDA announcements but by other online sources.

With the introduction of the BodyGuardian Remote Monitoring System from Preventice Solutions in 2012, the first FDA-approved AI/ML-based medical device was introduced^23^. This initiated further investments in innovations for the detection of cardiac rhythm abnormalities, resulting in 14 medical devices and algorithms for this purpose. The other two algorithms in this field focus on the detection of cardiac murmurs (eMurmer ID, CSD Labs GmbH). Interest from multinational technology companies is evident, with two FDA-approved algorithms from Apple Inc, being the ECG App and Apple Irregular Rhythm Notification Feature.

Application of AI/ML-based algorithms for rapid interpretation of the most general values in medical care, being the vital signs, was achieved by Excel Medical Electronics, Spry Health and Current Health. To further assist medical personnel in general, Stratoscientific, Inc. introduced the Steth IO device to analyse heart and lung sounds.

With two AI/ML-based algorithms, BrainScope Company Inc. has introduced AI/ML for the evaluation of brain injuries. At first, this company introduced Ahead 100, an electroencephalograph-based algorithm to evaluate patients after a mild traumatic brain injury. This algorithm was further developed, resulting in the introduction of BrainScope TBI, an algorithm which can be used for a broader scope of traumatic brain injuries—from functional abnormality (concussions) to structural injury (brain bleeds)^24^. Further attention goes to a gamified neurorehabilitation program, MindMotion GO (MindMaze SA), which is introduced to support rehabilitation for the elderly^25^. Using motion capture technology and an AI/ML-based algorithm, this invention promotes functional improvements. Other areas of interest are the assessment of memory loss in the eldery (Cantab Mobile, Cambridge Cognition Ltd) and seizure monitoring (Embrace, Empatica Srl.)^26,27^.

With a high disease burden and a shortage of care providers, the medical field of psychiatry is in need of AI/ML-based support^28^. Research efforts focus on the diagnosis and stratification of psychological disorders, followed by subsequent treatment support strategies. Two of these AI/ML-based algorithms reached the stage of FDA approval, QbCheck (QbTech AB) and ReSET-O (Pear Therapeutics Inc.). With QbCheck, healthcare workers can substantiate their diagnosis or rule out attention deficit hyperactivity disorder (ADHD), enhancing objective medical decisions in psychiatry^29^, whereas ReSET-O can be applied for patients with Opioid Use Disorder, providing cognitive behavioral therapy as a mobile medical application for prescription use only. As a next step, the ReSET-O algorithm will be used in a randomized controlled trial, which is scheduled to start this year (April, 2020)^30^.

## Discussion

In the beginning of the AI/ML era, it is especially important for the medical and scientific community to have a clear understanding of what medical technologies are considered AI/ML based and which ones are regulated to see how those can become elements of the toolset of medical professionals. Our database shows that it is possible to define the threshold for categorizing a technology as AI/ML based and also, that there is a need from regulatory agencies to create their own databases.

Authors of this study hope that this database can serve as a basis for the databases regulatory bodies will launch and the definition of threshold they will publish.

There are limitations to our approach. We aimed at determining the state of AI/ML-based medical devices and were met with three initial obstacles. (1) We chose to look only at regulated devices as companies tend to overhype the importance of their technology or simply use the terms AI and ML for the sake of more investments. (2) We chose to look at FDA approvals as the FDA has shown leadership in regulating AI/ML-based medical technologies and has published policies about that. And (3) we had to determine what should be considered as an AI/ML-based medical technology based on claims made in publicly available information sources.

While we aim to maintain the database along with contributions from the scientific community, the primary aim of the project is to encourage the FDA and other regulatory bodies to provide a clear overview of approved AI/ML-based medical devices and algorithms.

These obstacles also describe the possible limitations of our approach. First, not all AI/ML-based medical devices have been approved by the FDA, therefore we had to exclude many unregulated technologies from the database. Second, there are numerous AI/ML-based medical devices and algorithms in other countries outside the scope and supervision of the FDA. And third, as the FDA does not maintain such a database, we had to set the threshold for assessing what technology should be considered as AI based. It is possible that the terms and expressions we looked for could be too narrow for assessing AI/ML-based technologies.

While the scope of solving these issues exceeded our capacity, we believe that this paper represents the results of a pioneering work. No other curated database of FDA approvals of AI/ML-based devices and algorithms that serve medical purposes exists. As such solutions become increasingly adapted in the healthcare setting, the importance of such an easily accessible database will become more apparent.

While we aim to maintain the database along with contributions from the scientific community, we encourage the FDA and other regulatory bodies to take over this database or launch their own. The EMA already provides guidelines and statements about AI and would benefit from the addition of such a repository. This will help those local and international institutions and the public as the use of AI/ML-based medical technologies in healthcare has become imminent.

The FDA, which is the very source of these approvals, possesses the necessary resources required to perform in-depth analysis regarding the credibility and accuracy of algorithms for medical purposes. Especially, having access to the background database of their search engine where the content of FDA approval summaries is available.

Also, regulatory bodies could establish a clearer definition of what falls under the terms AI and ML This would also make it easier for developers, companies, researchers, journalists, and the general public to determine whether an approved technology is in fact AI/ML based.

We aimed at providing a method and a model for achieving that.

On top of the rise in AI/ML-based algorithms, reports show a comparable increase in the global market size for AI/ML in healthcare. The value is projected to soon exceed US$28 billion from its 1 billion value in 2016^31^. Software and devices using the technology will inevitably contribute to this market share.

However, despite the increasingly available AI/ML-based medical solutions on the market, there remains the factor of adopting those very solutions. The challenge to adopting these in the medical practice can be attributed to hindrances due to regulatory frameworks and trust issues with new technologies from both the physicians and patients side^32^.

There needs to be a paradigm shift when implementing new technologies, including AI/ML-based ones, in the healthcare system and key regulators play a crucial role in this as stated in the previous section.

According to our findings, there were no AI/ML-based devices approved by the FDA in 2010 and 2011. In the year 2012, we identified two while in 2019 alone, there were 22, amounting the number of relevant software programs to 64. In a decade, there was a major increase in the amount of these technologies.

As new approvals of AI/ML-based medical technologies become available, it will become even more difficult to keep track of all relevant announcements. This open access database that we set up and will continuously maintain could help serve as a reference for interested parties working on similar software or for research purposes; and as such will hopefully be a useful tool for the medical and scientific community.

## Methods

### Reasoning for not being able to do searches thoroughly on fda.gov

The most obvious method for our research would have been to check the official FDA database on FDA.gov and look for AI/ML-related terms in the summaries or approvals. The FDA’s official website does feature a search engine option, making all of its announcements freely accessible. However, searches cannot be filtered according to their content such as approval announcements or specific words. Looking for AI/ML-related terms in this way is not possible on the FDA’s search engine.

The FDA also provides reports of approvals in the 510(k), PMA and de novo categories on a monthly basis, but these provide basic information about the device or software approved and its company, with no mention of its function^33^.

In the next section, we outline the search strategy we chose to use that still ensures that all items in the database are cross-checked but could also mean that the database might not be complete. While not ideal, this issue also underscores the notion that the FDA’s website could improve its search function with a filter feature so as to provide more streamlined results. Moreover, given that the database we built is open to submissions, we hope to make it as complete and as comprehensive as possible.

### Search strategy employed for the database

For our search, we used a search strategy with the following steps: first, reliable resources, where FDA announcements appear regularly, were selected; second, these resources were used to identify relevant announcements; third, all identified announcements were cross-checked in the search engine of fda.gov.

**Fig. 2:**
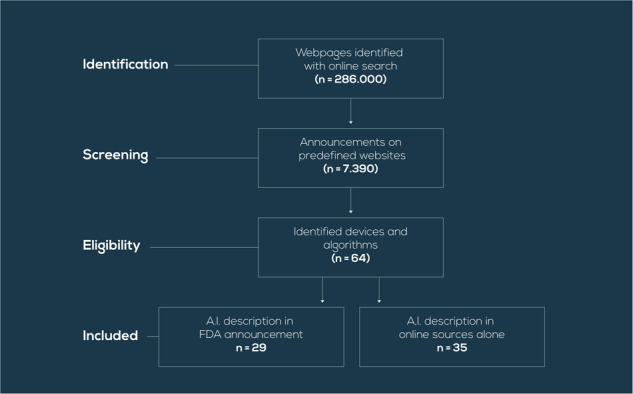
Flowchart for the selection of AI/ML-based algorithms for this online database, including the number of identified webpages, the number of screened announcements, the number of eligible devices and algorithms, and the final number of included AI/ML-based devices and algorithms.

Announcements from the websites listed were prioritized, scrutinized for relevant ones and then cross-checked on the FDA’s website for the official approval: mobihealthnews.com; bmj.com; medscape.com; lancet.com; sciencedaily.com; radiologybusiness.com; and acrdsi.org. In the announcements, we had to focus on key words that would point to the use of AI/ML in a medically oriented device or software that has been approved by the FDA (Fig. 2).

As the FDA does not have a threshold for marking devices as AI/ML- based, our observations showed that the most logical way to determine if a software program or device can be considered as AI/ML based is if the official FDA announcement contains any of these expressions: “machine learning”, “deep learning”, “deep neural networks”, “artificial intelligence”, and/or “AI.” Taking this into consideration, we used the following search strategy: (“FDA approval” OR “FDA clearance” OR “Approved by the FDA”) AND “Machine learning” OR “Deep learning” OR “Deep neural networks” OR “artificial intelligence” OR “AI”).

Nevertheless, we found that not all announcements use these terms even if the underlying algorithm can be defined as AI/ML based. For example, when looking for a specific device like AliveCor Inc.’s Kardia, the FDA announcement does not mention any of the expressions we looked for, while the company elsewhere states that their device uses an AI/ML-based algorithm^34^. We decided to include these devices and algorithms as well, although we clearly marked in the database that the related FDA announcements do not contain any of the expressions we looked for. Instead, we included those not FDA-related announcements with a URL that did.

We performed search queries for the time period between the 1st of January 2010 and the 1st of March 2020. As AI/ML-based technologies in medicine started gaining attention in the mid-2010s, we chose a longer time period to avoid missing important announcements. Once a relevant software program or product was identified from one of those websites, its authenticity was verified on fda.gov and recorded in our database with the accompanying FDA approval number.

We also extended our searches to other websites to ensure that we did not miss any announcements, but the majority came from the above-mentioned resources. Moreover, every single announcement recorded in our database was reviewed independently by the other authors of this paper.

### Online database and submission of new announcements

Following our collective input, cross-checking and validations, we created an online database to aggregate the results under the URL https://medicalfuturist.com/fda-approved-ai-based-algorithms/. This open access database will be regularly updated by the authors as the FDA approves new AI/ML-based devices and software.

The database includes the following details for each approval: name of the device or algorithm; name of the company; short description; FDA approval number; type of FDA approval; mention of AI/ML in the announcement, if no mention of AI/ML in FDA announcement (URL); date of approval; medical specialty it is related to; secondary medical specialty it is related to.

Due to the nature of the search strategy we chose to use, it is possible that we have missed some announcements. Therefore, we added a submission feature on the database, allowing the community to submit new ones that have been released after our search concluded or even those that we might have missed during our initial search. All community submissions will be cross-checked and verified before being considered to be added to the database.

### The infographic

In order to provide a clear picture about the state of AI/ML based, FDA-approved medical devices and algorithms; demonstrate which medical specialties those are related to, and how their number has been rising since 2010, we put all the cross-checked and verified devices in an infographic.

The infographic contains a device’s name, its short description, its relation to a primary and a secondary medical specialty and what kind of FDA approval it received. The same colors are assigned to the same medical specialty.

### Reporting summary

Further information on experimental design is available in the Nature Research Reporting Summary linked to this paper.

## Supplementary information

Reporting Summary Checklist FLAT

## Data Availability

The data that support the findings of this study are available on https://medicalfuturist.com/fda-approved-ai-based-algorithms.
